# A fault diagnosis method based on an improved diffusion model under limited sample conditions

**DOI:** 10.1371/journal.pone.0309714

**Published:** 2024-09-03

**Authors:** Qiushi Wang, Zhicheng Sun, Yueming Zhu, Dong Li, Yunbin Ma

**Affiliations:** 1 Key Laboratory of Networked Control Systems, Chinese Academy of Sciences, Shenyang, China; 2 Shenyang Institute of Automation, Chinese Academy of Sciences, Shenyang, China; 3 State Key Laboratory of Robotics, Shenyang Institute of Automation, Chinese Academy of Sciences, Shenyang, Liaoning Province, China; 4 PipeChina Institute of Science and Technology, Langfang, China; Beijing Institute of Technology, CHINA

## Abstract

As a critical component in mechanical systems, the operational status of rolling bearings plays a pivotal role in ensuring the stability and safety of the entire system. However, in practical applications, the fault diagnosis of rolling bearings often encounters limitations due to the constraint of sample size, leading to suboptimal diagnostic accuracy. This article proposes a rolling bearing fault diagnosis method based on an improved denoising diffusion probability model (DDPM) to address this issue. The practical value of this research lies in its ability to address the limitation of small sample sizes in rolling bearing fault diagnosis. By leveraging DDPM to generate one-dimensional vibration data, the proposed method significantly enriches the datasets and consequently enhances the generalization capability of the diagnostic model. During the model training process, we innovatively introduce the feature differences between the original vibration data and the predicted vibration data generated based on prediction noise into the loss function, making the generated data more directional and targeted. In addition, this article adopts a one-dimensional convolutional neural network (1D-CNN) to construct a fault diagnosis model to more accurately extract and focus on key feature information related to faults. The experimental results show that this method can effectively improve the accuracy and reliability of rolling bearing fault diagnosis, providing new ideas and methods for fault detection and prevention in industrial applications. This advancement in diagnostic technology has the potential to significantly reduce the risk of system failures, enhance operational efficiency, and lower maintenance costs, thus contributing significantly to the safety and efficiency of mechanical systems.

## Introduction

In a wide range of applications, such as industrial manufacturing, aerospace and automotive engineering, rolling bearings play a key and indispensable role. However, due to the complexity and variability of their operating environment, as well as possible improper maintenance and other problems, rolling bearings often become the most common failure components in rotating machinery [1]. As a core component in mechanical equipment, the health condition of rolling bearings has a profound impact on the performance and stability of the entire system [1, 2]. Therefore, timely and accurate fault diagnosis of rolling bearings is an indispensable part of ensuring the stable operation of mechanical equipment [3].

The process of fault diagnosis for rolling bearings typically encompasses a variety of methods and technologies. Currently, these methods and techniques can be systematically grouped into three main categories: model-based diagnostic methods, data-based analysis techniques [4], and hybrid integrated diagnostic strategies [5, 6]. Model-based diagnostic methods aim to simulate the actual running state and potential failure modes of bearings by constructing physical or mathematical models of the bearings, so as to realize fault prediction and accurate diagnosis of bearings in operation [7]. On the other hand, data-based analysis technology relies on bearing operating data collected by sensors in real time, and with the help of data analysis tools and pattern recognition algorithms, a comprehensive assessment of the bearing’s health status is carried out. However, with the increasing complexity of modern equipment, it has become increasingly difficult to construct models that can accurately reflect failure mechanisms, which to some extent limits the application of physical models in the field of fault diagnosis. Therefore, data-based fault diagnosis methods are currently favored as mainstream diagnostic techniques in practical applications due to their flexibility and practicality [8].

With rapid advancements in science and technology, the field of data-based fault diagnosis is experiencing unprecedented changes. With this wave of change, deep learning-based fault diagnosis methods have garnered significant interest and application [9, 10]. This is attributed to their ability to automatically extract and process features from raw vibration data, showing notable potential for practical application [11]. Guo et al. [12] improves the comprehensiveness and accuracy of fault diagnosis by fusing the time-domain and time-frequency-domain features of signals through parallel network deployment, while combining the anomalous attention mechanism of AT and the attributes of CBAM to form a dual attention mechanism. Chen et al. [13] proposed a bearing fault diagnosis algorithm based on multisource sensor data and an improved long short-term memory network (LSTM), which can effectively fuse features and cope with noise interference, improving diagnostic accuracy. Shao et al. [14] proposed a high-precision deep learning algorithm for machine fault diagnosis based on transfer learning, which converts sensor data into images, extracts features through pretrained networks, and fine tunes the network architecture. Chen et al. [15] combined CNN with transfer learning and proposed a transferable CNN algorithm that reuses prior knowledge to improve the learning performance of deep models in mechanical fault diagnosis. Xiao et al. [16] proposed a fault diagnosis algorithm based on a graph neural network (GNN). The algorithm constructs a graph through sample similarity, uses a GNN for feature mapping, fuses neighbor feature information, and then inputs the mapped samples into the basic detector for fault detection. Meanwhile, the attention mechanism, which has made a large splash in the field of natural language processing and computer vision, is now being actively explored and applied to the field of fault diagnosis by researchers, such as channel attention [17], spatial attention [18], self-attention [19], CBAM [20], and coordinate attention [21], which have led to new breakthroughs in fault diagnosis technology [22].

However, the above research relies heavily on laboratory environments where faults are artificially created to generate large amounts of fault data. Conversely, in real production environments, rolling bearings are shut down immediately when they fail, and companies tend to adopt preventive maintenance, which makes it difficult to collect fault data. In the realm of fault diagnosis [23], a large amount of normal data and a relatively small amount of fault data often occur during the monitoring process [24]. To solve this challenge, researchers have made many efforts and attempts. Yan et al. [25] proposed a deep regularized variational autoencoder (DRVAE) fault diagnosis method to optimize the VAE through regularization techniques, solve its overfitting problem, and enhance the feature learning capability of the model. Zhao et al. [26] proposed an improved generative adversarial network (GAN), which optimized the training process and improved the diagnostic performance by introducing auxiliary classifiers and autoencoder-based similarity estimation. Qiu et al. [27] proposed an auxiliary classifier generative adversarial network (ACGAN) to achieve controllable generation of category labels. Zhang et al. [28] proposed a CVAE-GAN model that enhances the GAN generator stability via a VAE encoder and introduces sample labeling to improve the training efficiency. When comparing the above methods, the GAN is deficient due to its instability in the training process and its susceptibility to pattern collapse, while the VAE is limited by the limited diversity of its generated data [29]. In contrast, a generative model called the denoising diffusion probabilistic model (DDPM) performs well in improving the quality and diversity of generated samples, and its training process is more stable and reliable. Cui et al. [30] proposed a fault diagnosis algorithm based on a symmetrized dot pattern (SDP) and DDPM, which converts one-dimensional vibration data into SDP and uses DDPM to generate samples to construct a datasets with significant and balanced features, thereby achieving accurate fault diagnosis. Yang et al. [31] generated more realistic and diverse generated samples based on DDPM and time-frequency maps of vibration data and mixed the real data with the generated data for fault diagnosis. However, methods using image data lead to the loss of temporal features when processing vibration data and lack additional guidance for the diffusion generation process.

Although significant results have been achieved in the field of small-sample fault diagnosis, considerable challenges remain in obtaining high-quality fault samples. For example, most of the current methods focus on sample generation from image data, while fault sample generation techniques for raw 1D data are still insufficient, which becomes a key challenge for us to further improve fault diagnosis performance. Therefore, this paper proposes an improved DDPM fault diagnosis method based on one-dimensional vibration data, aiming to solve the above problems and improve the diagnostic performance.

The contributions of this paper can be summarized as follows.

1. To address the problem of low model accuracy caused by insufficient fault data in rolling bearing fault diagnosis in reality, an improved 1D-DDPM model is proposed for generating fault samples.
2. The feature difference loss function is introduced in the training process of the 1D-DDPM model to make the generated data more directional and targeted and improve the quality of the generated samples.
3. Combining the data generation ability of the 1D-DDPM method and the feature extraction ability of the convolutional neural network, a 1D-DDPM-CNN fault diagnosis method is constructed, and the experiments show that this method is effective and accurate for the fault diagnosis of limited sample datasets.

The paper is organized as follows: The Methods section presents the methodology employed in this study. The Results and discussion section shows the results and discussion, and finally, the paper concludes with the Conclusion section.

## Methods

**Fig 1:**
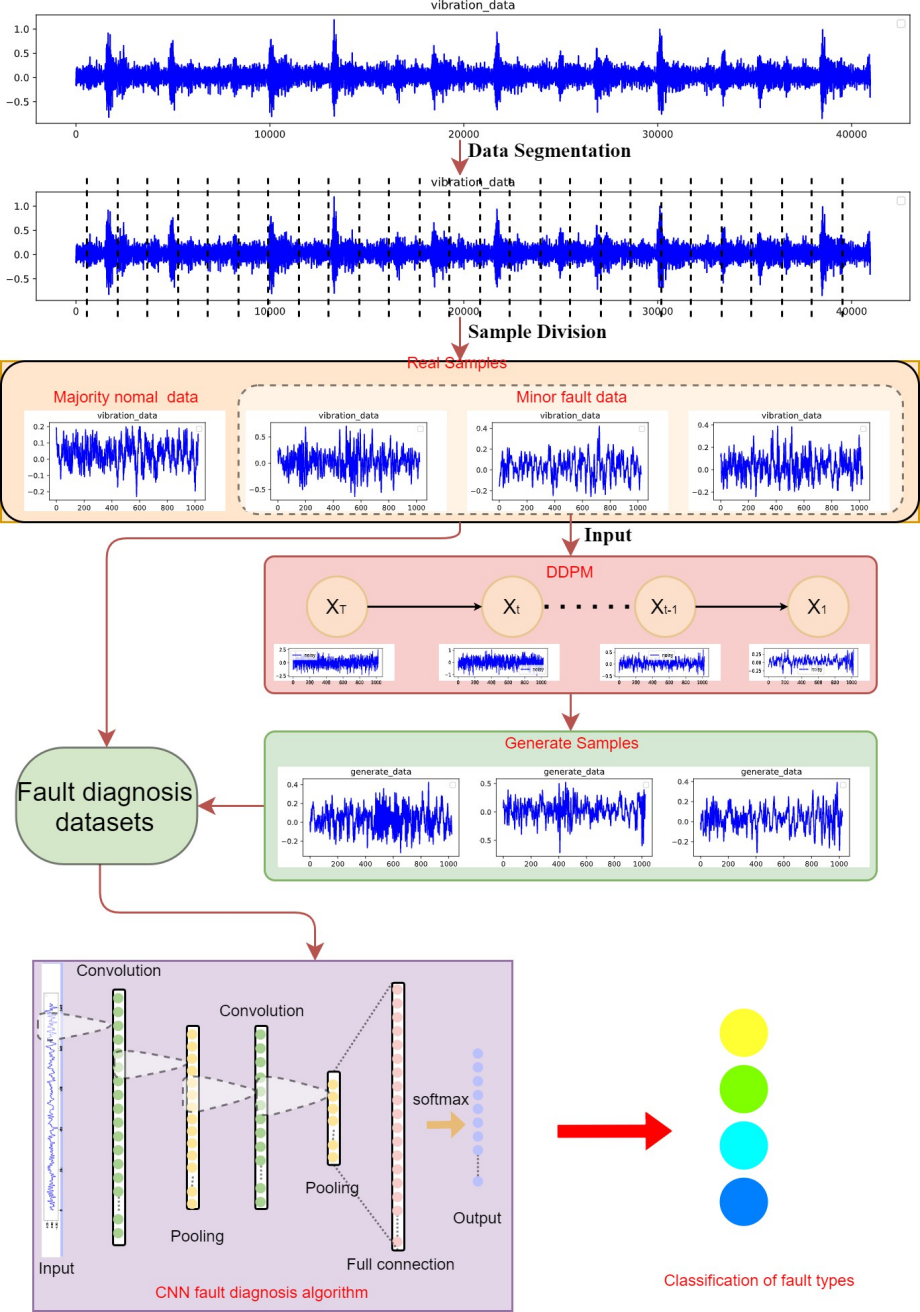
The structure of the fault diagnosis method.

This article proposes a rolling bearing fault diagnosis method that integrates one-dimensional DDPM and CNN, aiming to solve the problem of scarce fault data in real production environments. This method first uses one-dimensional DDPM to generate fault data, then mixes the original data with the generated data, and finally uses one-dimensional CNN for fault diagnosis. The specific process of the algorithm is shown in Fig 1.

### 1D-DDPM

The concept of diffusion modeling has been rooted in researchers’ exploration since 2015, and after several years of deep cultivation and sharpening, its theory and application have gradually matured. Until 2020, Jonhan Ho and other scholars successfully introduced the DDPM model on the basis of previous work and after subtle adjustments to the mathematical structure, which brought new innovative momentum to related fields. However, at present, the DDPM model mainly focuses on the generation of image data, and its application is still insufficient for the key area of fault diagnosis, especially fault diagnosis based on one-dimensional vibration data. As a direct carrier of equipment vibration information, raw 1D vibration data contain rich and detailed details. Therefore, this paper is dedicated to exploring the possibility of applying the DDPM model to 1D vibration data to utilize its unique advantages in the field of fault diagnosis.

**Fig 2:**
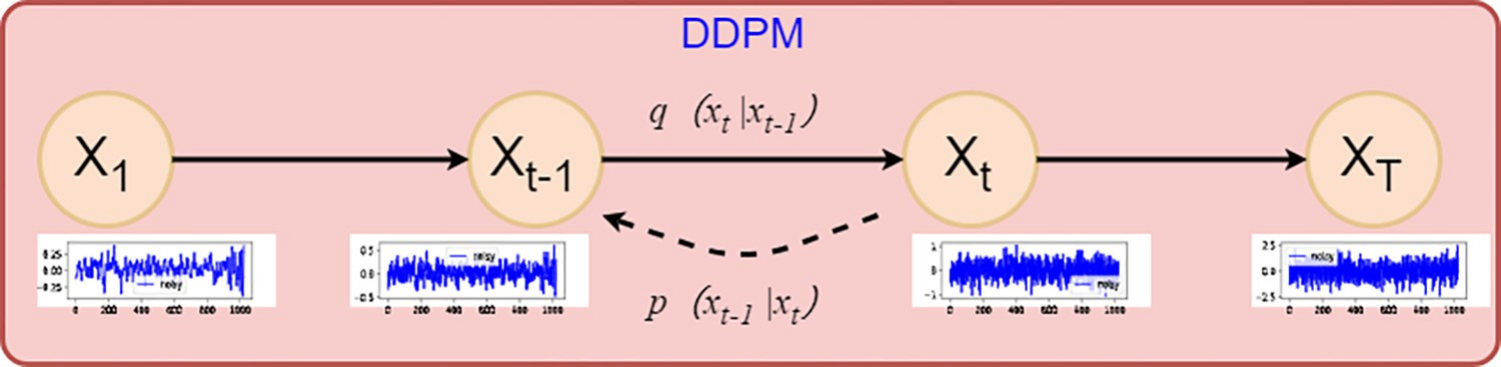
1D-DDPM schematic.

The structure of 1D-DDPM is shown as Fig 2.

As shown in Fig 2, 1D-DDPM mainly includes two processes: the forward process and the reverse process. The forward process involves gradually adding Gaussian noise to the original one-dimensional vibration data, ultimately resulting in the generation of pure Gaussian noise. The process of gradually adding noise is as follows:

$$x_{t}=\sqrt{1-\beta_{t}}x_{t-1}+\sqrt{\beta_{t}}z_{t}\;z_{t}∼N(0,I)$$ (1)

$$q(x_{t}|x_{t-1})=N(x_{t};\sqrt{1-\beta_{t}}x_{t-1},\beta_{t}I)$$ (2)

where x_0_ represents the raw vibration data, x_t_ represents the data after adding noiss, t represents the diffusion steps, β_t_ represents the diffusion rate, which gradually increases with increasing diffusion steps, z_t_ represents the Gaussian noise that conforms to the standard normal distribution, and N represents the Gaussian distribution.

In the forward diffusion process, x_t_ is related only to x_t-1_, so it can be regarded as a Markov chain process. For the convenience of calculation, let *α*_*t*_ = 1−*β*_*t*_ From this, it can be concluded that

$$x_{t}=\sqrt{{\bar{\alpha }}_{t}}x_{0}+\sqrt{1-{\bar{\alpha }}_{t}}z\;z∼N(0,I)$$ (3)

$$q(x_{1:T}|x_{0})=\prod_{t=1}^{T}q(x_{t}|x_{t-1})$$ (4)

The reverse process involves a gradual denoising procedure, entailing the step-by-step removal of noise from data adhering to a normal distribution, ultimately leading to the generation of one-dimensional vibration data.

The reverse process is the process of gradual denoising which gradually denoises noise data that conform to a normal distribution and generates one-dimensional vibration data. However, due to the need to determine the data distribution from the complete datasets, we cannot easily predict *q*(*x*_*t*_|*x*_*t*−1_). Therefore, the construction of a neural network parameterized by θ is adopted to approximate its distribution, assuming that *p*_*θ*_(*x*_*t*−1_|*x*_*t*_) is the probability distribution of the inverse process and obeys a Gaussian distribution with its mean *μ*_*θ*_ and variance ∑_*θ*_ both taking x_t_ and t as input parameters.


$$p_{\theta }(x_{0:T})=p(x_{T})\prod_{t=1}^{T}p_{\theta }(x_{t-1}|x_{t})$$ (5)

$$p_{\theta }(x_{t-1}|x_{t})=N(x_{t-1};\mu_{\theta }(x_{t},t),\sum_{\theta }(x_{t},t))$$ (6)

In the process of inverse diffusion, if we give x_t_ and x_0_, we can calculate x_t-1_ based on the posterior diffusion conditional probability.


$$q(x_{t-1}|x_{t},x_{0})=q(x_{t}|x_{t-1},x_{0})\frac{q(x_{t-1}|x_{0})}{q(x_{t}|x_{0})}$$ (7)

According to the properties of the Gaussian distribution and Formula (1), it can be concluded that

$$q(x_{t-1}|x_{t},x_{0})=N(x_{t-1};{\tilde{\mu }}_{t}(x_{t},x_{0}),{\tilde{\beta }}_{t}I)$$ (8)

Among them,

$${\tilde{\mu }}_{t}(x_{t},x_{0})=\frac{\sqrt{{\bar{\alpha }}_{t-1}}\beta_{t}}{1-{\bar{\alpha }}_{t}}x_{0}+\frac{\sqrt{{\bar{\alpha }}_{t}}(1-{\bar{\alpha }}_{t-1})}{1-{\bar{\alpha }}_{t}}x_{t}$$ (9)

$${\tilde{\beta }}_{t}=\frac{1-{\bar{\alpha }}_{t-1}}{1-{\bar{\alpha }}_{t}}\beta_{t}$$ (10)

According to Formula (3), it can be obtained that

$$x_{0}=\frac{x_{t}-\sqrt{1-{\bar{\alpha }}_{t}}z}{\sqrt{{\bar{\alpha }}_{t}}}\;z∼N(0,I)$$ (11)

By substituting it into Formula (9), it can be concluded that

$${\tilde{\mu }}_{t}(x_{t},t)=\frac{1}{\sqrt{\alpha_{t}}}(x_{t}-\frac{\beta_{t}}{1-{\bar{\alpha }}_{t}}z_{\theta })$$ (12)

*z*_*θ*_ in the formula is the added noise that needs to be predicted by the model.

The loss function of the original DDPM calculates the difference between the predicted noise and the true noise distribution. To make the generated model more interpretable and directional, this paper adds a sample quality evaluation loss to the loss function. By calculating the feature difference between the original vibration data and the predicted vibration data generated based on the predicted noise, the features are first normalized, and then the MSE loss is calculated. The selected features included time-domain indicators such as the mean, absolute mean, variance, standard variance, root mean square amplitude, root mean square value, peak, maximum, minimum, waveform index, peak index, pulse index, margin index, skewness, and kurtosis. To maintain sample diversity, the weights of the losses are 0.9 and 0.1, and the specific calculation method is as follows:

$$loss=0.9\times E_{x_{t},t,z}[{\left\|z-z_{\theta }(x_{t},t)\right\|}^{2}]+0.1\times E_{x_{t},t,z_{\theta }}[{\left\|y(x_{0})-y_{\theta }(x_{\theta }(x_{t},t,z_{\theta }))\right\|}^{2}]$$ (13)

where y represents the calculation function of the feature.

The specific calculation formulas for each feature are listed in Table 1.

**Table 1:** The specific calculation formulas for each feature.

1.mean $\bar{x}=\frac{1}{N}\sum_{n=1}^{N}x(n)$	2.absolute mean $|\bar{x}|=\frac{1}{N}\sum_{n=1}^{N}\left|x(n)\right|$
3.variance $\delta =\frac{1}{N}\sum_{n=1}^{N}x_{n}^{2}$	4.standard variance $\sigma_{x}=\sqrt{\frac{1}{N-1}\sum_{n=1}^{N}{\left[x(n)-\bar{x}\right]}^{2}}$
5.root mean square amplitude $x_{r}={\left(\frac{1}{N}\sum_{n=1}^{N}\sqrt{\left|x(n)\right|}\right)}^{2}$	6.root mean square value $x_{rms}=\sqrt{\frac{1}{N}\sum_{n=1}^{N}x^{2}(n)}$
7.peak $x_{p}=\max|x(n)|$	8.maximum $x_{p}=\max x(n)$
9.minimum $x_{\min}=\min x(n)$	10.waveform index $W=\frac{x_{rms}}{\bar{x}}$
11.peak index $C=\frac{x_{p}}{x_{rms}}$	12.pulse index $I=\frac{x_{p}}{\bar{x}}$
13.margin index $L=\frac{x_{p}}{x_{r}}$	14.skewness $S=\frac{\sum_{n=1}^{N}{\left[x(n)-\bar{x}\right]}^{3}}{(N-1)\sigma_{x}^{3}}$
15.kurtosis $K=\frac{\sum_{n=1}^{N}{\left[x(n)-\bar{x}\right]}^{4}}{(N-1)\sigma_{x}^{4}}$	

### 1D-CNN

**Fig 3:**
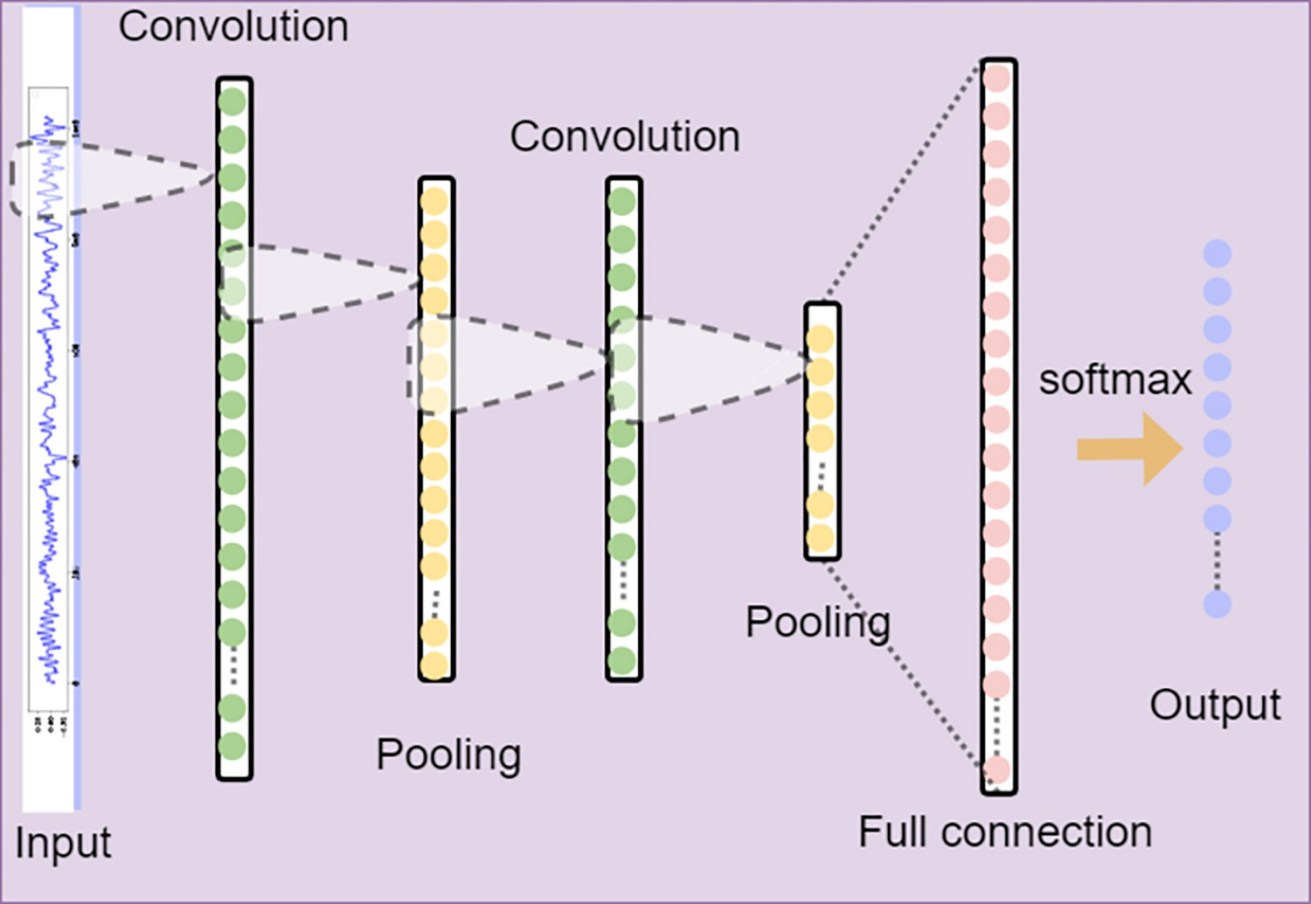
The structure of the 1DCNN.

One-dimensional convolutional neural networks (1DCNNs), as variants of convolutional neural networks, perform excellently in handling local relationships in sequence data. It can not only reduce the complexity of the model and avoid tedious feature extraction processes, but also effectively reduce the number of required weights. Therefore, this article specifically uses a 1DCNN to process one-dimensional vibration data, and its structural diagram is shown in Fig 3.

In this paper, the network structure of a 1DCNN is constructed by alternating convolutional and pooling layers, and finally, the extracted features are mapped to the output through a fully connected layer. In the convolutional layer, a series of trainable convolutional kernels slide over the input data to extract features, which can accurately capture the local patterns of the input sequence. Moreover, the application of activation functions results in nonlinear transformations on the output of convolutional layers, further enhancing the model’s expressive power. The convolution process can be described by the following formula:

$$y_{i,j,k}=f(\sum_{i=l}^{s}x_{i,k}*w_{j,i}+b_{i})$$ (14)

where y_i,j_, and k are the results of the convolution operation, f represents the ReLU activation function used in this article, x_i,k_ represents the input data, * represents the convolution operation, w_j,i_ represents the weights, and b_i_ represents the bias.

The pooling layer performs dimensionality reduction on the output of the convolutional layer, significantly reducing computational complexity and enhancing the robustness and generalization ability of the model. This article adopts the maximum pooling method, and the specific process is as follows:

$$X=\underset{(i-1)l+1\leq t\leq il}{max}X^{l-1}(t)$$ (15)

where X represents the feature map after dimensionality reduction and l represents the length of the pooling region.

Finally, the fully connected layer maps the output of the pooling layer to the final output of the model and identifies the output probability through the softmax function to complete the fault diagnosis task.

## Results and discussion

To comprehensively verify the effectiveness and superiority of the method proposed in this paper, we designed an exhaustive experiment. In this study, we choose two representative datasets for the experiments, aiming to comprehensively test the generalization ability and stability of the method. In addition, to objectively evaluate the performance of the method proposed in this paper, we conducted comparative experiments with popular generative models such as Variable AutoEncoder (VAE) and Generative Adversarial Network (GAE). We also compare CNN fault diagnosis algorithms that do not use generative models to highlight the advantages of this paper’s approach in dealing with data scarcity and generative capabilities.

For the hardware configurations of the experiments, we chose a high-performance computing environment, including a Windows 10 operating system, an RTX 3090 GPU, and a Core i7-12700K processor. These configurations provide sufficient computing resources for the experiments and ensure the accuracy and reliability of the results.

During the experiments, all the fault diagnosis models were trained iteratively for 100 training cycles, and a learning rate of 0.001 and an Adam optimizer were used for parameter optimization and model tuning to ensure that the models were fully trained and converged. We chose Python as the development language and relied on the powerful deep learning framework TensorFlow to build and train the models to ensure the smooth execution of the experiments and the accuracy of the results.

### Case 1: CWRU bearing datasets

#### Data description

The CWRU bearing fault diagnosis datasets is a commonly used datasets provided by Case Western Reserve University and is specifically designed for bearing fault detection and diagnosis. This datasets contains vibration signal data, covering the normal operating status of bearings and three common fault states, namely inner race faults, outer race faults, and rolling ball faults. These fault states simulate different fault situations that may occur in actual industry.

In this paper, we selected the 48k drive end bearing fault data from the CWRU bearing fault diagnosis datasets as the experimental data. Specifically, three types of faults were selected: inner ring fault, outer ring fault, and rolling element fault, with a speed of 1730 rpm, a horsepower of 3 hp, and a fault diameter of 21 miles. For the outer ring fault, we selected the fault data with the fault location at 12 o’clock. More descriptions of the datasets are described in Table 2.

**Table 2:** Description of the CWRU datasets.

Fault Type	Fault Size	Fault Position	Training Datasets For 1D-DDPM	Testing Datasets For FD model	Label
Inner Race Fault	21 mils	—	100	100	0
Ball Fault	21 mils	—	100	100	1
Outer Race Fault	21 mils	Centered@12:00	100	100	2
Normal	—	—	200	100	3

To construct the experimental data, we combined the fault data with the normal data. For each fault type, we randomly selected 200 samples as the datasets, each containing 1024 vibration data points. Among them, we use 100 samples as training samples for the generative model, and the other 100 samples as test datasets to evaluate the performance of the fault diagnosis model.For the normal type, as there is no need for data generation, 200 samples are randomly selected as training samples, and the remaining 100 samples are used as testing samples.

To solve the problem of difficult attribute data processing by classifiers, we adopted unique hot encoding instead of real number encoding. This encoding method converts each attribute value into a binary vector, where only one element is 1 and the rest are 0. This approach can effectively represent attribute data, enabling the classifier to better process and learn features.

Through such experimental design and data preprocessing methods, we can use the CWRU bearing fault diagnosis datasets for research on bearing fault detection and diagnosis. This will help us extract features related to faults and train models to automatically identify and classify different types of bearing faults.

#### The performance of the 1D-DDPM

The accuracy of fault diagnosis methods based on deep learning depends on the number of samples. The sample generation method of 1D-DDPM proposed in this paper can effectively supplement fault samples. However, the generated samples may be close to real samples in terms of their statistical characteristics, as they are actually generated through algorithm simulation, which cannot fully replicate the complexity and diversity of fault occurrence in the real environment and cannot completely replace real samples. To evaluate the impact of the number of generated samples on the accuracy of fault diagnosis, a series of experiments were conducted in this paper.

In these experiments, we first generated three types of fault samples using 1D-DDPM and mixed them with real samples to construct a training set. To ensure the consistency of the results, all data augmentation is performed only on the training set, while the test set contains only the original real samples. The purpose of doing so is to eliminate the interference of other factors when evaluating the impact of the generated sample size on accuracy. In addition, the number of normal samples used for training is not fixed but matches the number of faulty samples. This approach can ensure a balance between normal and faulty samples in the experiment, avoiding the impact of data imbalance on the results. Each experiment was repeated 10 times, and the average accuracy was recorded in Table 3.

**Table 3:** Diagnostic results of different generated samples.

Experiment number	Training samples (fault types)		Training samples (normal type)	Testing samples	Average accuracy (%)
	Real samples	Generate samples	Real samples		
1	600	0	200	400	98.68
2	450	0	150	400	95.73
3	300	0	100	400	93.52
4	150	0	50	400	90.35
5	75	0	25	400	88.78
6	300	600	300	400	97.92
7	300	450	250	400	99.53
8	300	300	200	400	97.08
9	300	150	150	400	95.23
10	300	75	125	400	93.82
11	0	600	200	400	91.53
12	0	450	150	400	94.14
13	0	300	100	400	88.73
14	0	150	50	400	80.12
15	0	75	25	400	74.28

In the above experiments, we conducted a series of different experiments, covering experiments 1 to 15. Experiments 1 to 5 considered only real data, experiments 6 to 10 used mixed generated data with real data for experimentation, and experiments 11 to 15 used only generated data for fault diagnosis.

From Experiment 1 to Experiment 5, it can be observed that the accuracy decreases as the number of training samples decreases. This indicates that the number of samples has a significant impact on accuracy without generating samples.

Through experiments 6 to 10, it can be observed that as the number of generated samples increases, the accuracy improves. However, when the ratio of generated samples to original samples is 1:1.5, the fault diagnosis accuracy is the highest. Excessive generation of fault data does not improve the accuracy of diagnostic models, as these samples may contain redundant information in addition to fault information. This further indicates that the generated samples cannot completely replace the real samples.

Through experiments 11 to 15, it can be observed that using only generated samples for training without using real samples resulted in a decrease in accuracy compared to experiments 1 to 5. This indicates that although generating samples can fit the distribution of real data, there are still certain limitations in fault diagnosis.

**Fig 4:**
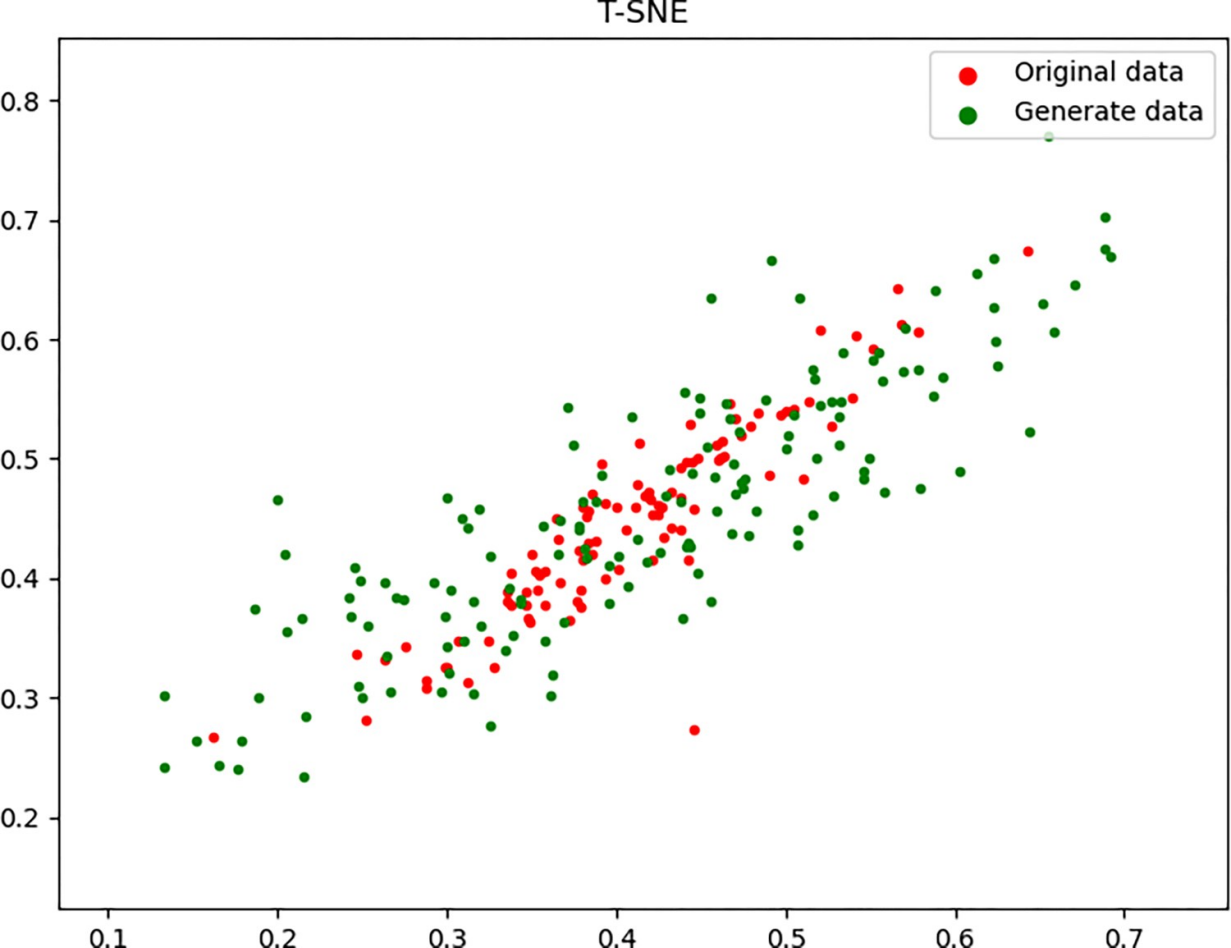
The gap between original data and generate data.

In summary, we can conclude that the method of mixing generated data with real data has better performance in fault diagnosis compared to a single data source. This means that mixing generated data with real data can provide better performance in fault diagnosis. However, although generating data can fit the distribution of real data well, there is still a certain gap in fault diagnosis compared to real data. This gap is mainly reflected in two aspects, namely, noise difference and dynamic characteristics. In terms of noise difference, although the generated data are similar to real data in distribution, they lack the randomness and complex noise in real-world data, which affects the robustness of the fault diagnosis model, and in terms of dynamic characteristics, the real data often contain complex dynamic processes, which are difficult to be fully captured by the generated model, leading to a diagnostic performance gap. The gap between the generated data and the original data can be visualized as Fig 4, which shows the original data is more centralized in distribution than the generate data.

The method of mixing generated data with real data performs well in improving fault diagnosis performance. When the ratio of real samples to generated samples is 1:1.5, the fault diagnosis effect is optimal. Although the sample generation method effectively compensates for the problem of insufficient samples, it is still necessary to pay attention to the situation where there is a certain gap in fault diagnosis performance between the generated data and the real data.

### Fault diagnosis of rolling bearings based on 1D-DDPM-CNN

To further evaluate the feasibility and effectiveness of the method proposed in this article, comparative experiments were conducted. First, we use different sample generation methods to generate fault samples, where the ratio of real samples to fault samples is 1:1.5. Next, we use a mixed datasets to train the fault diagnosis model. Table 4 describes the detailed information of the data.

**Table 4:** Mixed datasets of rolling bearing samples in different states.

Fault state	Training samples		Testing samples
	Real samples	Generate samples	
Normal	250	0	100
Inner-ring fault	100	150	100
Outer-ring fault	100	150	100
Rolling-ball fault	100	150	100

Through the above experimental setup, we evaluate the impact of different sample generation methods on fault diagnosis models.

In this paper, a fault diagnosis model is constructed based on a 1D-CNN, and the hyperparameter settings are shown in Table 5.

**Table 5:** The hyperparameters settings.

Kernel counts (KC)	Kernel length (KL)	Pooling Length (PL)	Dropout	Learning Rate	Batch Size
32	32	3	0.2	0.001	16

**Fig 5:**
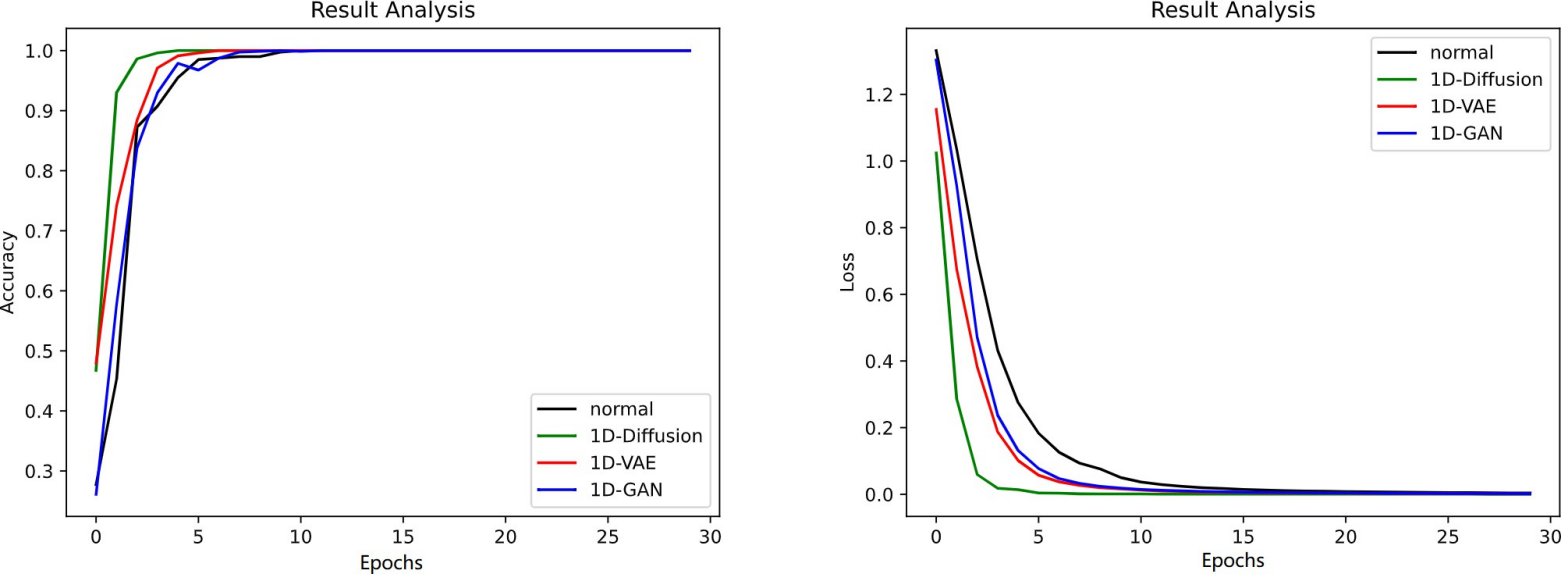
The accuracy and loss of the fault diagnosis methods.

The number of convolution kernels for the 1D-CNN in this article is set to 32, and the length of each convolution kernel is set to 32. When performing pooling operations, use a pooling window of size 3 is used. To reduce overfitting, the dropout is set to 0.2, which will randomly discard a portion of the neurons in the network. The initial learning rate is set to 0.001, and after every 5 iterations, the learning rate is halved. The batch size is set to 16 and the number of epochs is 30. Categorical_crossentropy is used as the loss function to update the model parameters. **Fig 5** shows the training results of fault diagnosis for different generative models.

By observing the figure, it can be observed that after 10 iterations, the training accuracy and loss of the four methods reached a convergence state, and the accuracy all reached 100%. However, compared to algorithms that only use real samples, algorithms that mix three types of generated samples with real samples have faster convergence speed and higher accuracy. In particular, the 1D-DDPM algorithm used in this article performs the best among these methods, proving the effectiveness and superiority of our method.

Table 6 shows the accuracy, recall and F1 values of the four fault diagnosis methods on the test set. From the table, it can be seen that the method proposed in this paper performs the best on all three indicators, further proving the effectiveness of the method proposed in this paper.

**Table 6:** The result of the four fault diagnosis methods using CWRU.

Fault diagnosis model	1D-DDPM-CNN	1D-VAE-CNN	1D-GAN-CNN	1D-CNN
Accuracy	99.75%	97%	96%	95.25%
Recall	99.752%	97.04%	96.02%	95.53%
F1	99.751%	97.02%	96.01%	95.39%

**Fig 6:**
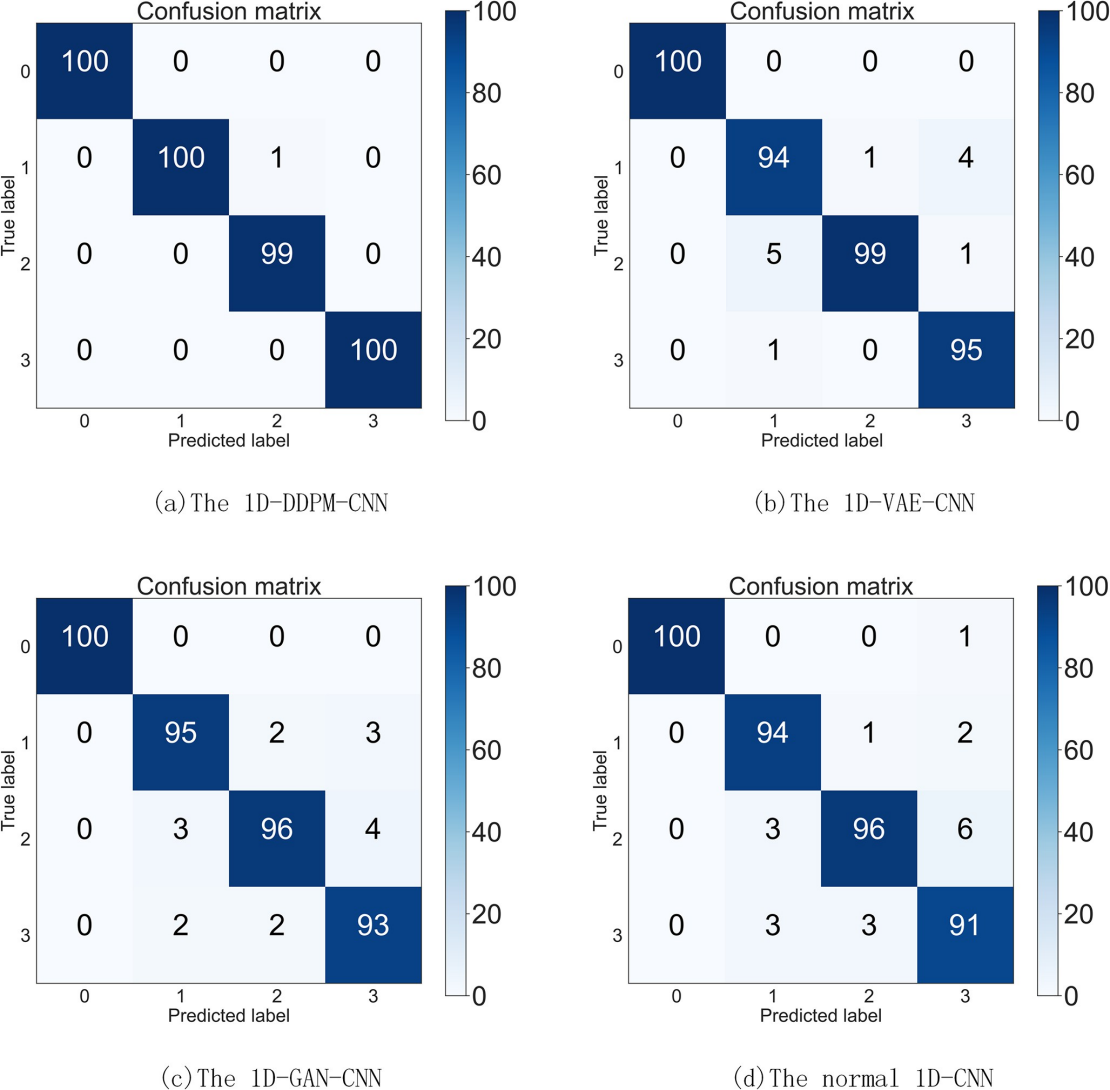
The confusion matrix of the different algorithms.

To further validate the effectiveness of the developed method, we plotted **Fig 6**, which shows the confusion matrix obtained when using a CNN model for fault diagnosis after supplementing fault samples with three generation models namely, 1D-DDPM, 1D-VAE, and 1D-GAN, and mixing them with real samples. The y-coordinate of the confusion matrix represents the classification of the actual labels, and the x-coordinate represents the predicted labels. The main diagonal elements of the confusion matrix represent the number of correctly classified samples in the current category,and the diagnostic accuracy for each running state is shown in Table 7.

**Table 7:** The accuracy for each operation condition of different algorithms.

Fault diagnosis model	1D-DDPM-CNN	1D-VAE-CNN	1D-GAN-CNN	1D-CNN
IF	99%	95%	95%	94%
OF	100%	99%	96%	96%
BF	100%	94%	93%	91%
Normal	100%	100%	100%	100%

The recognition accuracy of the four algorithms for normal samples is 100%. However, for the fault samples generated by mixing the generated samples with the real samples, the recognition accuracy did not all reach 100%. This indicates that although the generative model can effectively supplement the missing fault samples, there is still a gap in the data quality of the generated samples compared to the real samples. On the other hand, for the recognition accuracy of the three types of fault samples, the algorithm proposed in this paper shows the highest accuracy, further proving the effectiveness and superiority of the algorithm proposed in this paper.

### Case 2: JNU bearing datasets

#### Data description

The JUN bearing datasets, which originated from Jiangnan University, encompasses a comprehensive collection of bearing running status data [32]. This datasets was generated utilizing a centrifugal fan system test bed, equipped with a Mitsubishi SB-JR induction motor, where a fault was intentionally introduced into one of the bearings. The accelerometers were positioned perpendicular to the bearings to capture the vibration signals. The datasets encompasses four distinct running states: normal, inner ring fault, outer ring fault, and rolling element fault. The vibration acceleration signals were precisely captured at a sampling frequency of 50 kHz, across various rotational speeds of 600, 800, and 1000 rpm, providing a rich resource for multivariate analysis. For this study, the four states at 600 rpm were selected for fault diagnosis experiments, and the specific details are outlined in Table 8.

**Table 8:** Description of the JNU datasets.

Fault Type	Training Data-sets For 1D-DDPM	Testing Data-sets For FD model	Label
Normal	0	250	0
Outer Ring Failure (OF)	100	250	1
Rolling Body Failure (BF)	100	250	2
Inner Ring Failure (IF)	100	250	3

#### Fault diagnosis of rolling bearings based on 1D-DDPM-CNN

**Fig 7:**
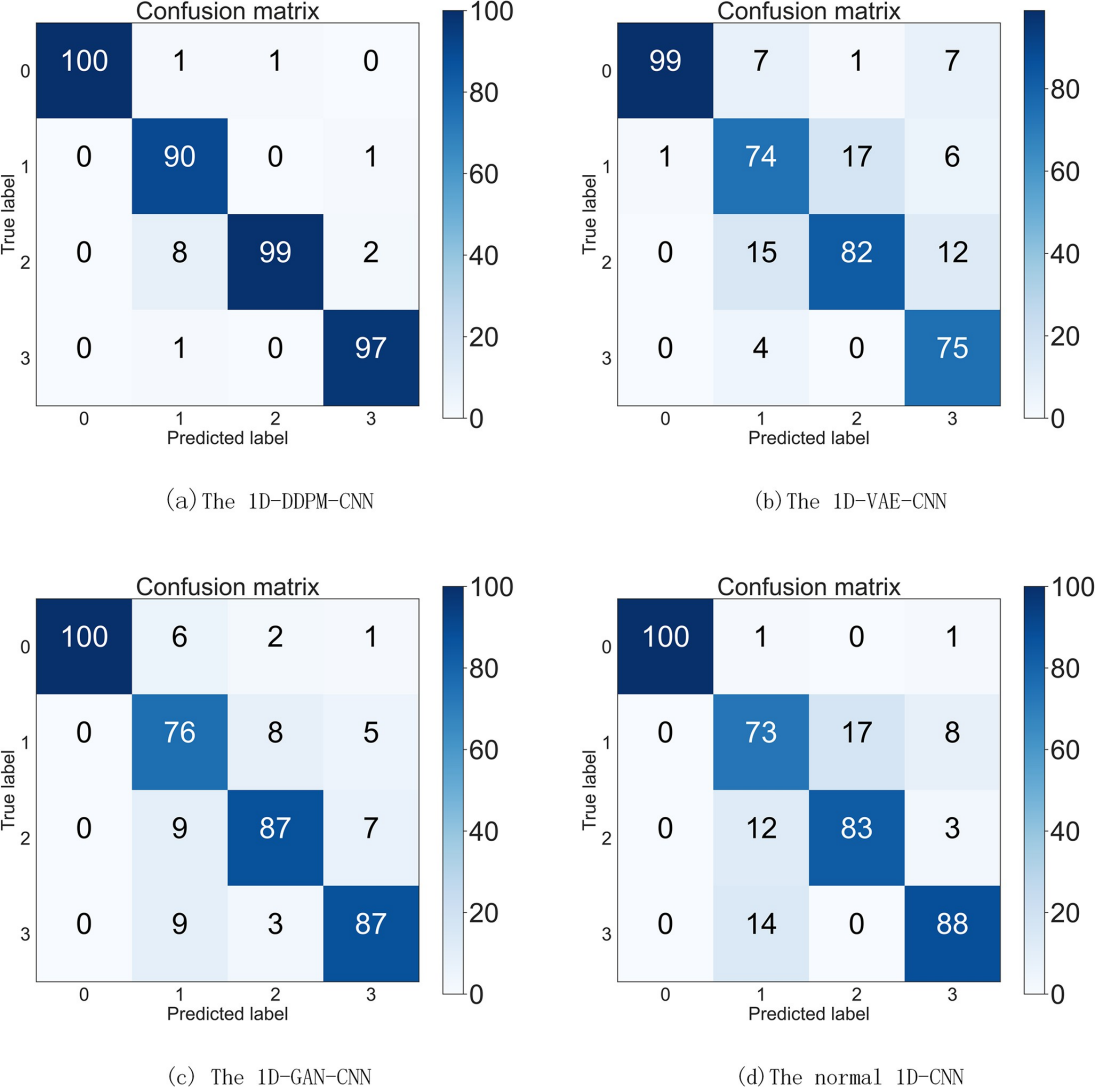
The confusion matrix of different algorithms in JNU datasets.

The optimal ratio was verified by the CWRU datasets, i.e., a 1:1.5 ratio of real data to generated data. We generated 150 simulated data samples for each of the three different fault types based on 100 real data samples, and added an additional 250 normal data samples, which together constructed a comprehensive fault diagnosis training set. For the test set, we ensure that each operation state contains 100 samples to comprehensively evaluate the model performance. In this experiment, we tested the 1D-DDPM-CNN model, the VAE-CNN model, the GAN-CNN model proposed in this paper, and a traditional CNN model without using generated samples. The experimental results are shown in Table 9, and the confusion matrix of the results is shown in **Fig 7**. The results show that the algorithm proposed in this paper achieves the optimal performance in terms of accuracy, recall, and F1, which fully verifies the efficiency and practicability of this algorithm in fault diagnosis tasks.

**Table 9:** The results of the four fault diagnosis methods using JNU.

Fault diagnosis model	1D-DDPM-CNN	1D-VAE-CNN	1D-GAN-CNN	1D-CNN
Accuracy	96.5%	87.5%	86%	82.5%
Recall	96.68%	87.37%	85.87%	83.73%
F1	96.59%	87.43%	85.93%	83.11%

#### Comparison with other methods

To further evaluate the performance of the proposed method and its effectiveness in the field of fault diagnosis, we conduct a comparative study with five existing and representative methods. These comparative methods include two generative models (i.e., DCGAN and ERGAN) and three nongenerative models (DTL-Res2Net-CBAM, SMOTE, and DCNN). During the experiments, we use the widely recognized CWRU datasets to ensure the reliability and wide adaptability of the experimental results. For the generative models, we selected 100 real samples and the corresponding generative samples for each fault type to jointly construct the training set, in contrast to the training set for the nongenerative models, where we used only 100 real samples. The test set used another 100 real samples for each type to evaluate and compare the performances of all the models. The experimental results are shown in **Table 10**.

**Table 10:** Comparison between fault diagnosis methods.

Fault diagnosis model	Accuracy
The proposed method	**99.75%**
DTL-Res2Net-CBAM	99.68%
ERGAN	99.34%
DCGAN	98.95%
SMOTE	97.98%
DCNN	97.81%

The experimental results show that the accuracy of the proposed method in this paper is the highest for both generative and nongenerative models, further proving the effectiveness of the method proposed in this paper.

## Conclusion

The rolling bearing fault diagnosis method based on the improved denoising diffusion probability model (DDPM) proposed in this article has achieved significant results under limited sample conditions. By using DDPM to generate one-dimensional vibration data, the problem of insufficient data has been effectively solved, and the generalization ability of the model has improved. At the same time, introducing feature differences into the loss function makes the generated data more directional and improves diagnostic accuracy. In addition, the CNN model can better capture key features and enhance the robustness of the model.

However, there are still some shortcomings in this study. Under extremely sparse sample conditions, the performance of this method has not been fully validated, and its adaptability needs to be further strengthened. Moreover, this method also has certain limitations in terms of model transfer performance, making it difficult to directly apply to the fault diagnosis of rolling bearings of different models or working environments.

In future work, we will focus on addressing the issues mentioned above and implementing the following recommendations. First, we will dedicate efforts to optimizing the model structure, enhancing its universality, and improving its generalization ability. This will enable the model to effectively adapt to various fault modes in a better manner. Second, we will explore fault diagnosis methods specifically designed for situations with extremely sparse sample conditions. By doing so, we aim to enhance the model’s performance in such scenarios. Additionally, we will investigate strategies to improve the transfer performance of the model, allowing it to be applied more extensively in different scenarios for fault diagnosis of rolling bearings.
